# Contrastive Learning-Based Anomaly Detection for Actual Corporate Environments

**DOI:** 10.3390/s23104764

**Published:** 2023-05-15

**Authors:** Gi-taek An, Jung-min Park, Kyung-soon Lee

**Affiliations:** 1 Korea Food Research Institute, Wanju-gun 55365, Republic of Korea; gt@kfri.re.kr (G.-t.A.); parkjm@kfri.re.kr (J.-m.P.); 2 Division of Computer Science and Artificial Intelligence, CAIIT, Jeonbuk National University, Jeonju 54896, Republic of Korea

**Keywords:** enterprise information system dataset, anomaly detection, contrastive learning, constructing dataset

## Abstract

Information systems play an important role in business management, especially in personnel, budget, and financial management. If an anomaly ensues in an information system, all operations are paralyzed until their recovery. In this study, we propose a method for collecting and labeling datasets from actual operating systems in corporate environments for deep learning. The construction of a dataset from actual operating systems in a company’s information system involves constraints. Collecting anomalous data from these systems is challenging because of the need to maintain system stability. Even with data collected over a long period, the training dataset may have an imbalance of normal and anomalous data. We propose a method that utilizes contrastive learning with data augmentation through negative sampling for anomaly detection, which is particularly suitable for small datasets. To evaluate the effectiveness of the proposed method, we compared it with traditional deep learning models, such as the convolutional neural network (CNN) and long short-term memory (LSTM). The proposed method achieved a true positive rate (TPR) of 99.47%, whereas CNN and LSTM achieved TPRs of 98.8% and 98.67%, respectively. The experimental results demonstrate the method’s effectiveness in utilizing contrastive learning and detecting anomalies in small datasets from a company’s information system.

## 1. Introduction

Enterprise information systems not only support and manage company-wide resources efficiently but are also the core information technology (IT) and SW systems that encompass a company’s business innovation activities. Operating a company without the help of information systems has become impossible. According to the IT and SW utilization survey conducted by the Korea Institute for Information and Communications Technology Promotion in 2018, 93.5% of Korean companies have introduced and utilized enterprise resource planning (ERP), while 93.8% of companies have not adopted ERP owing to their business environment and characteristics [1].

Information systems play an important role in business management, including personnel, budget, and financial management. In the case of an abnormality in the information system, all operations are paralyzed until its recovery. To ensure the stable operation of important information systems, companies designate relevant departments and system administrators and make efforts to respond to abnormal situations through monitoring. However, the complexity of information systems makes it difficult to maintain and respond quickly to abnormal situations. In addition to system monitoring, information system administrators in companies are responsible for system management and related tasks, such as system improvement and administrative work.

In monitoring systems, notification functions, such as those for email and text messages, are available and can be customized based on predefined thresholds and situation settings. However, configuring specific situations for monitoring systems can be challenging because the settings may need to be adjusted based on the situation and environment unique to a company. Experienced monitoring system experts may struggle to set up accurate notifications if they are unfamiliar with a company’s setup. Setting the threshold for notifications considerably high can result in failures occurring before they are detected, whereas setting them considerably low can lead to unnecessary notifications. Due to these challenges, companies usually identify system failures only after they have occurred.

Deep learning models have been widely used for detecting abnormal states, and deep learning-based research on abnormal state detection in corporate information systems includes research into IT infrastructure and information systems. The detection of abnormal states in IT infrastructure involves detecting the abnormal states of devices such as servers and networks, whereas the detection of abnormal states in information systems aims to detect the abnormal states of web services. Depending on the perspective, different data features are considered important, and the data composition for learning differs. Abnormal states in information systems may also occur as abnormal states in IT infrastructure, and therefore both should be considered together.

In order to apply anomaly detection in corporate environments, it is necessary to use data from real operating systems. However, to use data from a company’s information system, methods for merging independently produced data into a deep learning dataset are required, and the data needs to be labeled to train the system to detect anomalies.

In this study, we propose a deep learning-based anomaly detection method using monitoring data from company information systems. We collected data from an information system for 338 days, from 28 September 2021, to 31 August 2022. Regarding data from information systems operated by companies, it is difficult to secure anomalous data as the stable operation of the information system cannot be compromised. In addition, even if data are collected over a long period, the composition of the training dataset may have more normal data than anomalous data. To address the problem of data imbalance, we propose the use of negative sampling, and to improve training performance with insufficient data, we propose a data augmentation method for contrastive learning. The proposed method contributes to the utilization of actual operating system data for anomaly detection in corporate environments. In addition, our proposed method can further enhance the effectiveness of existing monitoring software.

The rest of this paper is structured as follows: Section 2 presents related work; Section 3 describes the construction of the dataset for anomaly detection in information systems; Section 4 describes contrastive learning with the negative sampling method; Section 5 presents the experimental results; and Section 6 concludes the paper.

## 2. Related Work

Anomaly detection research focuses on IT infrastructure and information systems. In the case of IT infrastructures, the aim is to identify abnormal states in servers and networks, whereas in information systems, the goal is to detect abnormal states in web services. These two domains differ primarily in their perspectives, which may lead to differences in the collected data.

### 2.1. Anomaly Detection in IT Infrastructure

The detection of abnormal states in an IT infrastructure involves the use of server metrics such as CPU usage, memory usage, and network response time. In addition to these performance metrics, data such as service response time and processing volume are employed to detect abnormal states in information systems. Anomalies in IT infrastructure can lead to failures in information systems; hence, there is a need to monitor both domains simultaneously.

Several deep learning-based approaches have been used for anomaly detection in IT infrastructure. Spectral residuals [2] and an ensemble model have been used to analyze time-series access log data in real time, using data such as access count, response time, CPU usage, and memory usage to predict response delays [3]. Unsupervised learning and a convolutional neural network (CNN) [4] have been combined to improve the labeling of difficult abnormal data, leading to better performance compared to that achieved when using only spectral residuals. A CNN and log data from the Hadoop file system have been used to achieve superior performance over that of long-short-term memory (LSTM) [5] and MLP models through automatic log analysis [6]. Finally, using support vector machine (SVM) [7] models, researchers experimented with preprocessing and vision processing techniques, with preprocessing-based learning ultimately proving more effective for anomaly detection.

There have been several investigations carried out to detect anomalies within diverse IT infrastructures utilizing time-series data. For instance, Shukla and Sengupta [8] focused on identifying anomalous states within clustered sensors using an LSTM neural network and robust statistical M-estimators. Ngo et al. [9] applied adaptive anomaly detection to a distributed hierarchical edge computing system for real-time detection on devices that cannot use complex deep neural networks. Using the wavelet autoencoder anomaly detection technique, Li and Jiang [10] utilized an autoencoder to detect anomalous data in non-stationary and non-periodic time-series data. In addition, Chang et al. [11] proposed a hierarchical anomaly detection framework to distinguish between real and fake data to detect various malicious intrusions on IoT devices. Yin et al. [12] proposed a model that combines CNN and a recurrent autoencoder, using a two-stage sliding window method for better feature extraction. Talagala et al. [13] proposed an unsupervised learning algorithm for anomaly detection in high-dimensional data that used the distribution of k-nearest neighbors.

In studies on anomaly detection applications for IoT sensors, some studies extracted contextual information to predict the contextual information of the system [14], while others detected anomalous states through two stages using clustering and fuzzy logic [15].

In research focused on identifying anomalies in network and communication systems, there have been studies such as AnoML-Iot [16], Del-Iot [17], a low-weight model that employed blockchain technology [18], and an investigation that detected indications of anomalies in HTTP traffic [19]. All of these studies employed time-series data and applied lightweight techniques or used edge technology for IoT models.

### 2.2. Anomaly Detection in Information Systems

There have been several research investigations into utilizing deep learning-based anomaly detection methods for information systems. Two commonly used models are the LSTM and autoencoders. In one LSTM-based study that employed the LSTM model, the emphasis was on forecasting service quality based on web service response time and processing volume. The data classification was performed using techniques such as PCA and tSNE, and the LSTM model outperformed [20] other models such as the decision tree, AdaBoost [21], multilayer perceptron, XGBoost [22], LightGBM [23], and CatBoost [24]. In another study that used PCA and weblog data, PCA and KNN algorithms were implemented for detecting anomalies [25].

In studies utilizing autoencoders, regular data were evaluated by adding noise patterns to generate abnormal data. Shin et al. [26] constructed a conditional multimodal autoencoder that outperformed unimodal and multimodal models. Lee [27] used a deep autoencoder for anomaly diagnosis in a database management system (DBMS) and demonstrated its potential for automatic DBMS diagnostic reports.

In studies utilizing multivariate time series data, Audibert et al. [28] used DNN-based methods to detect anomalies. Additionally, Schmidl et al. [29] conducted a recent evaluation study comparing 158 methods for anomaly detection, which included models such as LSTM-based VAE-GAN [30], LSTM-VAE [31], LSTM-AD [32], Spectral Residual (SR), SR-CNN, RobustPCA [33], AutoEncoder (AE) [34], Bagel [35], and EncDec-AD [36]. The study concluded that simpler methods perform almost as well as more complex ones.

In studies utilizing contrastive learning, both image data [37,38] and graph data [39,40,41] have been used. Since contrastive learning incorporates data augmentation, it is predominantly applied in research areas where data augmentation can be easily carried out. Similarly, research in the field of anomaly detection is mainly concentrated on areas where data augmentation is more viable.

Previous research has focused mainly on developing anomaly detection models and evaluating their effectiveness. Studies conducted using data resembling enterprise environments have used data from companies with large internet data centers. However, obtaining big data to train deep learning models is challenging for typical enterprises, and replicating similar studies in real-world scenarios is difficult due to differences in information system infrastructure across companies.

The objective of this study is to develop an anomaly detection model for information systems using deep learning techniques. To achieve this, we collected data from the operating system of a company’s information system to construct a dataset. In addition, we incorporated feature data that had been previously used in anomaly detection research. Finally, we compared the proposed method with CNN and LSTM models.

## 3. Dataset Construction

**Figure 1:**
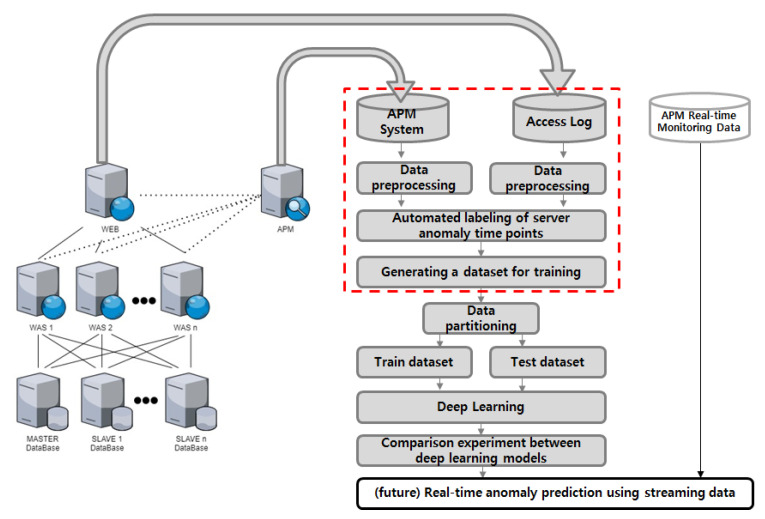
Process of constructing a deep learning dataset for anomaly detection in actual information systems.

In order to construct a deep learning dataset for anomaly detection, we gathered data from our company’s operational information system, which includes one IIS-based web server and four Tomcat-based web application servers. We developed a monitoring system to collect performance information from the system. We collected access log data from the web server and performance monitoring data from the monitoring system to create a deep learning dataset for anomaly detection. The process of collecting and processing data from an actual enterprise information system is depicted in Figure 1.

Labeled data is required to detect anomalous states using deep learning. In this study, the anomalous states were systemically labeled based on expert observations using the collected data, resulting in 48,333 data frames.

### 3.1. Collecting Data from the Performance Monitoring System

The performance monitoring system provides diverse information on the servers and web application servers to assess the performance of the information system. Moreover, the collected monitoring data could be extracted as separate data and utilized for further analysis. In this study, we employed the open-source software ScouterAPM (2.15 Version) for performance monitoring. ScouterAPM enables the checking of different web programs and database indicators based on Java and has the advantage of being open source, which makes it easily applicable and usable for businesses.

**Figure 2:**
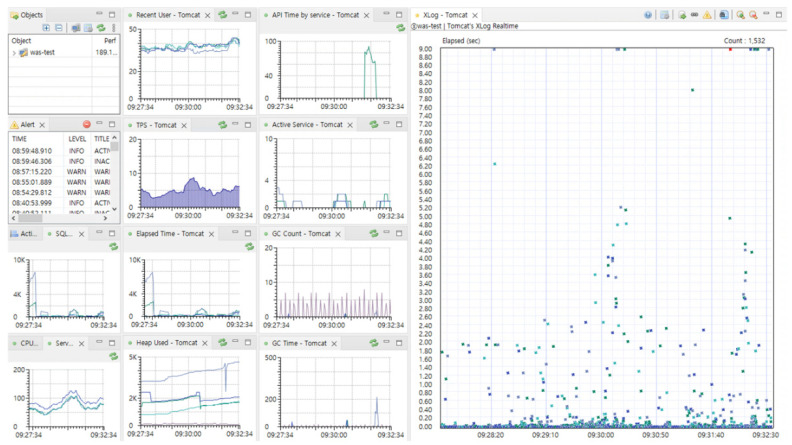
Monitoring dashboard of ScouterAPM, an open-source performance monitoring software. Ten types of performance monitoring data (lines) and response time based on user requests (dots).

Figure 2 displays the operational dashboard of ScouterAPM, which can monitor a range of indicators for web application servers, including CPU and memory information of servers that run the information system, as well as various metrics of the web application server. The system also allows the storage of monitored performance metrics.

In this study, we selected all relevant metrics that could affect the occurrence of anomalies in the information system, and we chose 10 performance monitoring metrics. We collected performance monitoring data for 338 days. Table 1 summarizes the collected data, which comprises 389,376 rows.

The features collected by the performance monitoring system can be categorized as follows: items 1–6 are indicators that affect the performance of the web server, whereas items 7–9 are indicators that affect the memory performance of the web application server. Item 10 is an indicator that describes the performance of the database. Ten performance monitoring data points were collected from the four servers operated by the company.

**Figure 3:**
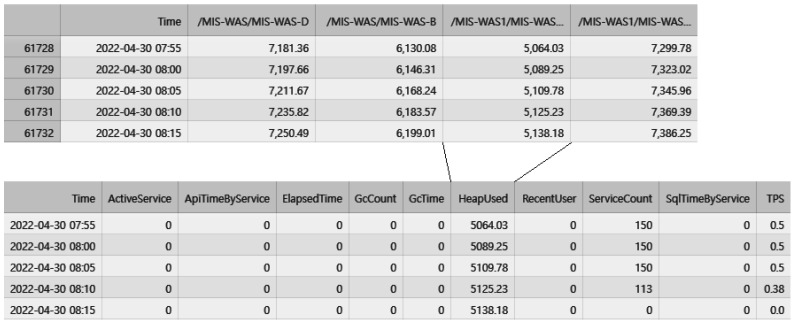
Transforming individual performance monitoring data into one dataset based on time.

**Figure 4:**
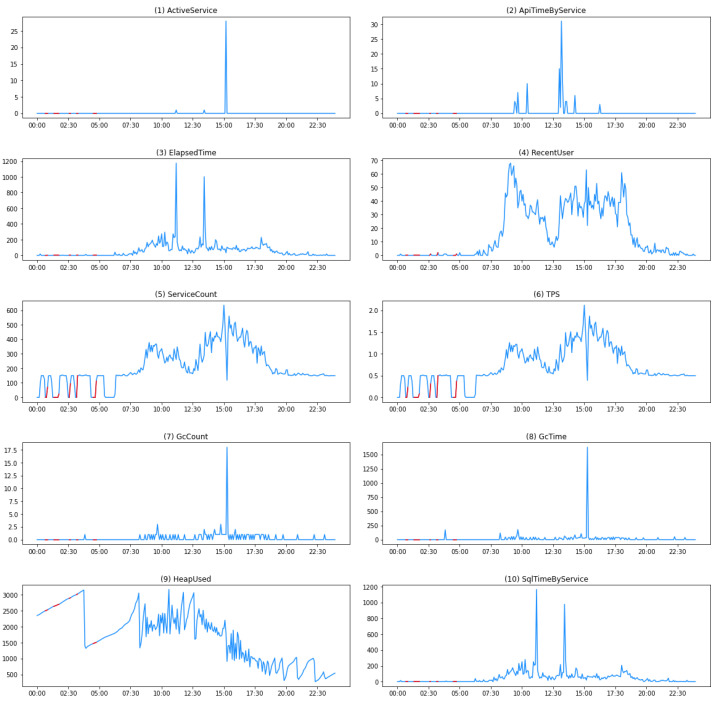
Visualization of performance monitoring data: 10 indicators. Note that the *y*-axis scale of each plot varies. The red segments indicate abnormal periods.

The collected performance monitoring data are in the form of time-series data at 5-min intervals. Each dataset includes server-specific values for the performance indicators. To detect anomalies, the 10 features are combined to create a time-series dataset. These datasets are merged into a single dataset based on the time required for deep learning. Figure 3 illustrates the process of transforming individual performance monitoring data into a single dataset based on time, and Figure 4 presents a visualization of the 10 performance monitoring data indicators.

All the data collected in this study are in the form of time-series data. Anomalies in the enterprise’s information system cannot be identified by a single indicator. For instance, a delay in the response time using the ElapsedTime feature alone does not necessarily indicate a problem with the system. This is because the enterprise’s information system may involve complex operations or various data joins, resulting in longer completion times, which does not signify an anomaly. Similarly, a temporary drop-in service count does not necessarily indicate an anomaly. There may be times when many users do not use the system, such as during lunchtime or departmental meetings, which is a normal occurrence. Therefore, we collected various data features to utilize in our research. In Section 3.3, the data collected in this section will be transformed into a suitable form for deep learning, along with the web server access log data collected in Section 3.2. Typically, data needs to be segmented into specific intervals or forms for deep learning purposes, and when solving classification problems with supervised learning, each data point needs to be labeled, as we conducted in our study.

### 3.2. Data Collection from Web Server Access Log

Load-balancing methods are commonly used in information systems to ensure high availability. Health check functions play a crucial role in this regard by monitoring the operating status of servers. One such health check method sends packets regularly to a specific port on a web application server connected to the web server and calls the URL to check the service status of the server, thereby examining any abnormal state. In this study, we collected data utilizing access logs to check for abnormal server states using a URL.

The advantage of confirming health check logs by URL calling is the ability to detect momentary service disruptions, and cases in which the server did not respond are recorded. To label the data, we checked the access logs of the web application server to see if there was any record of “GET/HTTP/1.1” being called with the response being 200, which indicates a normal state. If the call was not received, it was recorded as an abnormal state. Although access logs are recorded in text format, they contain multiple pieces of information, such as date, time, calling method, address, and response status, in a single line. Therefore, regular expressions were used to separate the data.

**Figure 5:**
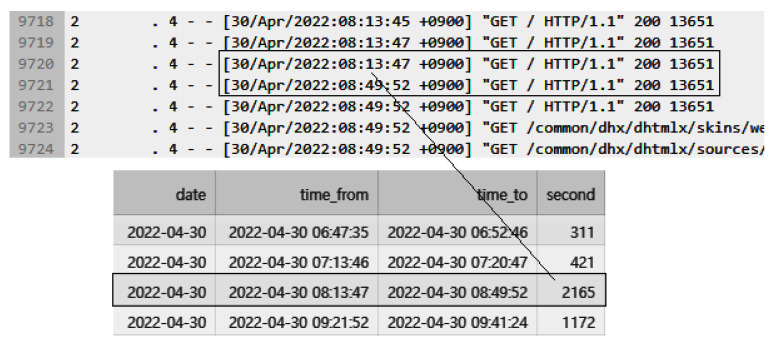
Transforming access log data into labeled data for detecting abnormal states.

Figure 5 illustrates the absence of response records for 2165 s between 8:13:47 and 8:49:52, which was used to determine the occurrence time of the anomalous state. The anomalous time interval, along with the previously collected performance data, was used to automatically label and construct a dataset for anomaly detection.

### 3.3. Automatic Labeling of Collected Data

In order to construct data for deep learning, it is necessary to label normal and abnormal states in the performance data. Typically, expert judgments and reports that record actual abnormal states are required to identify and label the abnormal states in the data. However, in this study, we were able to label the data automatically using a health check log.

**Figure 6:**
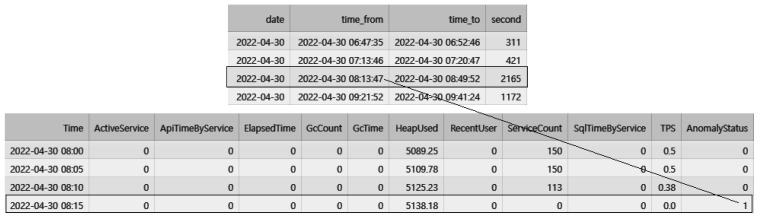
Transforming access log data into labeled data for detecting abnormal states.

In Figure 6, it can be observed that an anomaly occurred from “2022-04-30 08:13:47” to “2022-04-30 08:49:52” (“Year-Month-Date hours:minutes:seconds” format), lasting for a total of 2165 s. Based on this data, the period from “2022-04-30 08:15,” which is 13 min after the anomaly started, until the end of the anomaly at 49 min was labeled as 1 to indicate the anomaly in the transformed performance monitoring data. The same labeling method was applied to all other anomalies. To indicate a normal status, data that did not contain any anomalies was labeled as 0.

After reviewing the labeled anomaly data, we found that anomalies occurred frequently during the early morning hours owing to backup, server batch program operations, and other factors that caused a delayed response. Since detecting anomalies during business hours, when employees use the system, is important to the company’s information system, we only used data from 7:00 am to 8:00 pm. Consequently, we obtained a deep learning dataset consisting of 210,600 rows.

When constructing abnormal state data from performance monitoring data, it is crucial to consider the time-series nature of the data, which were collected at 5 min intervals. To ensure causality, 20 min of data were used to construct the abnormal state data, including 15 min of data prior to the occurrence of the abnormal state. Additionally, to match the size of the abnormal data, normal data were constructed using 20 min of data.

## 4. Contrastive Learning with Negative Sampling Method

Additionally, to conduct comparative experiments, we utilized established models such as CNN and LSTM as well as our own approach of contrastive learning with negative sampling. In all cases, we augmented the training data using negative sampling.

### 4.1. Convolutional Neural Network (CNN)

A CNN is a deep learning algorithm with two types of layers: convolutional and pooling layers. It offers the advantage of not requiring manual feature extraction and has demonstrated exceptional performance in domains such as image, video, and speech recognition.

In our experiment, a CNN learning model for anomaly detection was constructed using two convolutional, two pooling, and two fully connected layers. To prevent overfitting, a dropout rate of 0.01 was applied. As the input data were 10 × 4 × 1, padding was applied to maintain the data size as they passed through the layers. Additionally, this paper utilized 50 epochs, a batch size of 10, a learning rate of 0.001, the Adam optimizer, and the cross-entropy loss function.

### 4.2. Long Short-Term Memory (LSTM)

LSTM is an extension of the recurrent neural network proposed to address the long-term dependency problem. It comprises four gates that determine whether to retain or forget the previous information input through the Forget Gate, thereby enabling the model to remember and utilize past data. In contrast to recurrent neural networks, LSTM has demonstrated good performance in processing natural language and time-series data. To use LSTM, the data must be transformed into a sequential format before being fed into the model.

In our experiment, we transformed the collected time-series data into a matrix format with a 10 × 4 structure that can be further transformed into a 40 × 1 format. We constructed a classification model using an LSTM architecture that can accept 40 features as input and produces two results after processing the input. Our experiment utilized 50 epochs, a batch size of 10, a learning rate of 0.0001, the Adam optimizer, and the cross-entropy loss function.

### 4.3. Data Augmentation and Contrastive Learning

Contrastive learning is a self-supervised method in the field of machine learning that involves learning by comparing input samples. The primary objective is to construct a representation space where similar data points are positioned closely while dissimilar ones are placed far apart. By doing so, the model can learn a representation space that captures the distinctive features of each data point, resulting in improved performance on new tasks [42].

Contrastive learning utilizes data augmentation techniques to generate additional data and improve its generalization ability on new data by repeatedly applying various transformations to the input data and comparing the generated data to find similar data points. This approach enables the model to learn more generalizable features. In contrast to previous self-supervised learning methods that were developed from unsupervised learning, contrastive learning defines comparison targets from the training data, which makes it easier to implement self-supervised learning. This innovative learning method has demonstrated high performance in recent computer vision research [42].

Incorporating data augmentation in contrastive learning is common practice; however, it may exacerbate data imbalance problems, especially when dealing with real-world data where there are fewer anomaly data points.

**Figure 7:**
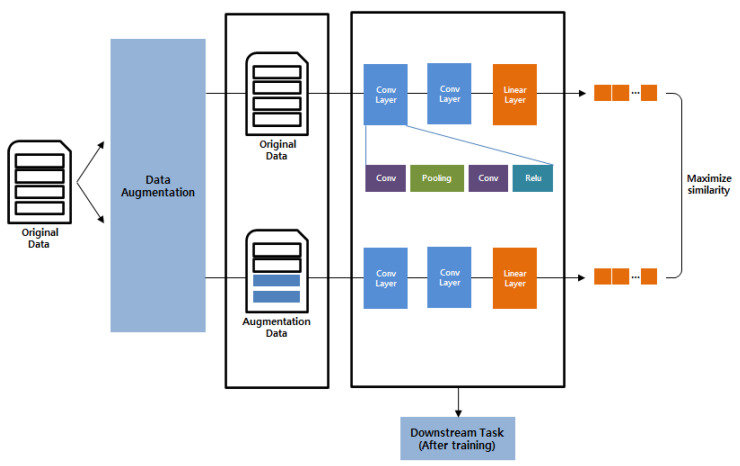
The architecture of the contrastive learning model.

Furthermore, to address this problem, this study employs data augmentation in contrastive learning to generate similar data for training, as illustrated in Figure 7. Moreover, negative sampling is utilized to balance the normal and anomaly data and is used as input data to tackle the imbalance problem. Both the original data and the augmented data go through the convolution layer, consisting of two convolutions and one max-pooling, with ReLU as the activation function. The model learns to represent similar data points closely and dissimilar data points far apart by calculating similarity based on the representations obtained from the two data sets using the loss function from SimCLR. The trained model undergoes downstream learning by being applied again to the existing CNN model and fine-tuned to create a single learning model. Our system employs 50 epochs, a batch size of 10, a learning rate of 0.001, the Adam optimizer, and the cross-entropy loss function.

In contrastive learning, both the original and augmented data are used as inputs for comparison. Various methods can be applied for data augmentation in the field of image or video processing, and data transformations, such as rotation, reduction, enlargement, or cropping, do not affect data identification.

However, for the data used in our experiment, the meaning of each value is crucial. Furthermore, although the order of the feature values may not be significant, if the feature values are swapped, the meaning of the data can change. Therefore, we utilize Algorithm 1 as the data augmentation method for contrastive learning. We merged the 10 min period before the anomaly occurrence, which was assumed to be a normal state, with the 10 min period of normal state data in close proximity to the anomaly occurrence to generate augmented data.
**Algorithm 1.** Abnormal Data Augmentation Algorithm**Input:** Normal Dataset is N, Abnormal Dataset is AbN **Output:** Augmented Dataset 1: for i from 0 to length of N 2:  newArr ← AbN [i] 3:  newArr [0] ← N [0] 4:  newArr [1] ← N [1] 5:  Arg[i] ← newArr


## 5. Experiment and Results

### 5.1. Experimental Environment

The performance of the proposed model was evaluated through a 10-fold cross-validation, with 90% of the data used for training and 10% for testing. The experiments were repeated 10 times, and the results of each repetition were averaged. The experiments were conducted on a computer with an Intel Xeon E5-2670 CPU (2.30 GHz) and an NVIDIA GeForce RTX 2080 Ti.

In terms of evaluating the performance of the comparative models, the cases for identifying the anomaly state are presented in Table 2, where true positive (TP) indicates the correct identification of an actual anomaly state, true negative (TN) represents the correct identification of an actual normal state, false positive (FP) refers to the incorrect identification of a normal state as an anomaly state, and false negative (FN) represents the incorrect identification of an anomaly state as a normal state.

Six evaluation measures were employed to assess the experimental results, including accuracy (ACC), true positive rate (TPR), false positive rate (FPR), precision, recall, and F1 score. ACC reflects the proportion of all correctly classified states, while TPR represents the percentage of actual anomalies that were accurately predicted, and FPR represents the percentage of actual normal states that were incorrectly predicted as anomalies. Precision is defined as the ratio of true positives to the total number of positive predictions, while recall is the ratio of true positives to the total number of actual positives. The F1 score is the harmonic mean of precision and recall. Equation (1) provides the definitions of these measures.

ACC: (TP + TN) / (TP + FP + FN + TN) TPR: TP / (TP + FN) FPR: FP / (FP + TN) Precision: TP / (FP + TP) Recall: TP / (FN + TP) F1 Score: 2 × (Precision × Recall) / (Precision + Recall)(1)

In the context of a company’s information system, the accurate detection of anomalous states is of higher priority than mistakenly classifying normal states as anomalous. This is because failing to detect the actual anomalous states poses a greater risk than misclassifying normal states. Thus, in this study, we emphasize the importance of TPR as a critical measure for evaluating a model’s ability to correctly identify anomalous states.

### 5.2. Performance Comparisons

We conducted experiments to compare the performance of CNN and LSTM models with our proposed contrastive learning model for anomaly detection. Specifically, we conducted experiments with and without negative sampling to evaluate the significance of applying this method. Table 3 presents the experimental results with negative sampling, whereas Table 4 presents the results without negative sampling.

The experimental results presented in Table 3 and Table 4 reveal that the incorporation of negative sampling improves the TPR and F1 scores. All CNN, LSTM, and the proposed contrastive learning model with negative sampling achieved an ACC greater than 98%, demonstrating high classification performance. With regards to TPR, which was the main emphasis of the study and measures the ability to correctly detect anomalies, the CNN, LSTM, and contrastive learning models with negative sampling achieved performance levels of 98.80%, 98.67%, and 99.47%, respectively. Based on these results, negative sampling has been proven to be effective for CNN, LSTM, and contrastive learning models.

The proposed model achieved an ACC of 99.16%, a TPR of 99.47%, a FPR of 1.16%, and an F1 score of 99.16%. Furthermore, the proposed approach surpassed the CNN and LSTM models with negative sampling in all the evaluation metrics. The experimental results indicate that the proposed data augmentation technique for contrastive learning and the dataset construction method are effective in detecting anomalies in information systems.

**Figure 8:**
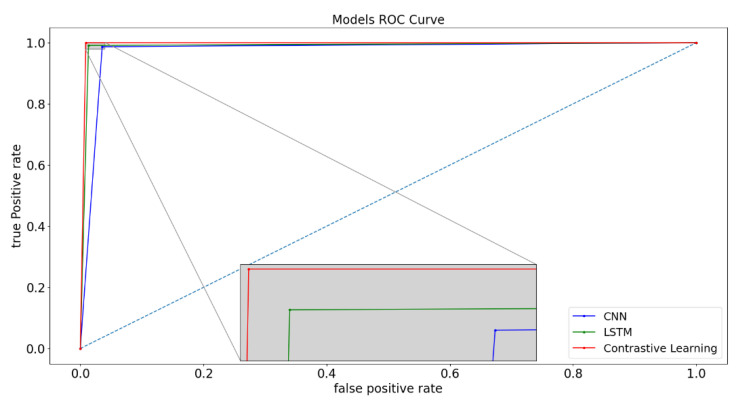
The ROC curves for the CNN, LSTM, and proposed Contrastive Learning Model. (The blue dotted line represents the reference line, while the gray line represents the magnified image of a particular point).

The presented ROC curves in Figure 8 demonstrate the high classification performance of the experimental models. Among the three models, our proposed contrastive learning method exhibited the most superior performance, while the LSTM model showed relatively lower performance.

### 5.3. Discussion

The detection of system anomalies in real-world business environments is crucial because system administrators often handle various tasks simultaneously and may not detect anomalies immediately. In this paper, we propose a method to address the data imbalance issue in real-world settings by using a performance monitoring system for data collection, negative sampling to overcome the challenge of collecting insufficient anomaly data, and contrastive learning in numerical data to achieve good performance with limited data. Firstly, we propose a data collection method that can be easily implemented by companies to gather training data for deep learning models that suit their specific environment. Secondly, as our experimental results show, using negative sampling to balance the data distribution between anomalous and normal instances leads to better performance than using imbalanced data for training. Finally, we experimentally confirmed that the data augmentation method for contrastive learning can be applied to numerical data and achieve good performance.

We applied our proposed method to deep learning models, including contrastive learning models, and compared their performance to commonly used models such as CNN and LSTM. Our experiments show that satisfactory results can be achieved through proper data processing; however, we believe that better sampling methods could further improve performance. We also tested the spectral residual-CNN model for anomaly detection [4], but it is not included in our results. However, anomalies were not detected in the data collected for this study, possibly due to disparities in usage patterns between actual companies and the system usage patterns at IDC.

Our study is particularly significant because there are relatively few studies that focus on a company’s information system, which is critical for its operations. By implementing our proposed method as a module for anomaly detection in a company’s monitoring system, data can be collected for learning, and a more user-friendly and practical function can be provided compared to the current alert systems. This can greatly aid in the operation of a company’s system.

## 6. Conclusions

In this paper, we propose a deep learning approach for detecting anomalies in real-world corporate environments. To address the challenge of imbalanced data between normal and anomalous states, we proposed a contrastive learning method with negative sampling. Additionally, we presented a data augmentation technique that can achieve high performance even with limited training data. Enterprise information systems are critical for managing personnel, budgets, and financial resources, and malfunctions can have a significant impact on business operations. Despite having designated departments and system administrators to monitor and respond to anomalies, the complexity of these systems makes it difficult to detect and respond to them quickly.

In order to address this challenge, we collected 338 days of actual operational data from enterprise information systems and proposed a contrastive learning approach that can detect anomalous states in information systems before or immediately after they occur. We used negative sampling to address the data imbalance issue and generated augmented data by modifying less influential points while maintaining the anomalous state. The proposed approach achieved high performance with ACC, TPR, and FPR scores of 99.16%, 99.47%, and 1.16%, respectively, outperforming CNN and LSTM models. The proposed method can be integrated into an enterprise’s monitoring system to collect data for learning and provide a more user-friendly and practical function than the current alert system.

The deep learning model we proposed in this study can be integrated into an APM (application performance management) system to detect anomalies in real-time data. Additionally, a supplementary system can be developed to continuously collect real-time data and alert system administrators when anomalies occur. This approach would be more advanced than the current monitoring system and provide a significant advantage for companies in maintaining system operations.

In future research, we can explore differentiating significant feature data to identify anomalies in information systems, conduct comparative analysis of new deep learning models, and investigate real-time data processing for anomaly detection.

## Figures and Tables

**Table 1:** Ten types of data collected through the performance monitoring system.

No.	Item	Description
1	ActiveService	Running service count
2	ApiTimeByService	API time by service
3	ElapsedTime	Average response time
4	RecentUser	Recent request user
5	ServiceCount	Number of service calls per hour
6	TPS	Transactions processed per second
7	GcCount	Garbage Collection operation count
8	GcTime	Garbage Collection time
9	HeapUsed	Heap memory usage
10	SqlTimeByService	SQL execution time by service

**Table 2:** Confusion matrix for performance evaluation.

	Actual	Abnormal	Normal
Predicted			
**Abnormal**		TP	FP
**Normal**		FN	TN

**Table 3:** Experiment Results of CNN, LSTM, and Contrastive Learning without Negative Sampling. (Bold:Best performance).

Metric	CNN	LSTM	Contrastive Learning
ACC	0.9897	0.9913	**0.9957**
TPR	0.9191	0.9048	**0.9333**
FPR	0.0068	0.0043	**0.0011**
Precision	0.8681	0.9097	**0.9749**
Recall	0.9191	0.9048	**0.9333**
F1 Score	0.8929	0.9073	**0.9536**

**Table 4:** Experiment Results of CNN, LSTM, and Contrastive Learning with Negative Sampling. (Bold:Best performance).

Metric	CNN	LSTM	Contrastive Learning
ACC	0.9858	0.9860	**0.9916**
TPR	0.9880	0.9867	**0.9947**
FPR	0.0164	0.0147	**0.0116**
Precision	0.9836	0.9853	**0.9885**
Recall	0.9880	0.9867	**0.9947**
F1 Score	0.9858	0.9860	**0.9916**

## Data Availability

The data presented in this study are available on request from the corresponding author. The data are not publicly available due to their containing confidential information related to the security of the company.
